# Survey of clinicians on the use of adjuvant therapy for premenopausal women with breast cancer

**DOI:** 10.1371/journal.pone.0290174

**Published:** 2023-08-17

**Authors:** Young-Won Lee, Sei-Hyun Ahn, Young-jin Lee, Tae-Kyung Yoo, Jisun Kim, Il Yong Chung, Hee Jeong Kim, Beom Seok Ko, Jong Won Lee, Byung Ho Son, Sae Byul Lee

**Affiliations:** 1 Division of Breast Surgery, Department of Surgery, University of Ulsan College of Medicine, Asan Medical Center, Seoul, Republic of Korea; 2 Department of Surgery, Ewha Womans University College of Medicine, Ewha Womans University Mokdong Hospital, Seoul, Republic of Korea; Local Health Authority Caserta: Azienda Sanitaria Locale Caserta, ITALY

## Abstract

**Purpose:**

Considering prognostic and anatomic stages in early-stage premenopausal patients with breast cancer, clinicians decide on performing the multigene assay, adjuvant chemotherapy, or ovarian function suppression (OFS). This decision is also based on genetic information related to hormone receptor-positive and human epidermal growth factor receptor 2 negative results. We aimed to determine the tendency to use adjuvant therapy in clinical practice.

**Methods:**

From April to May 2022, clinicians of the Korean Breast Cancer Society responded to a web-based survey. The survey included 62 multiple-choice questions mainly on decision-making under different pathologic conditions.

**Results:**

Among 92 responding clinicians, 91.3% were breast surgeons. For 35-year-old patients (pT2N0 and Ki-67 50% profile), 96.8% of clinicians selected chemotherapy, whereas 50.7% selected chemotherapy for patients with pT1N0, Ki-67 10%, and without Oncotype Dx (ODX). Only 35.6% selected chemotherapy for 47-year-old patients with the same profiles, while 84.3% and 49.1% chose chemotherapy with ODX recurrence score 21 and 16, respectively. More clinicians selected tamoxifen (TMX) plus OFS than aromatase inhibitor (AI) plus OFS for 5 years of endocrine therapy in patients with adjuvant chemotherapy regardless of genomic and clinical risks. However, for the same patients without adjuvant chemotherapy, more clinicians selected AI plus OFS. A longer duration of additional OFS and TMX was selected in patients with high clinical and genomic risks, and the duration of OFS was relatively shorter in older patients.

**Conclusion:**

The decision regarding adjuvant therapy should be made considering clinical and genomic risks and age, and clinicians should consult with patients about adverse effects and compliance.

## Introduction

At the turn of the century, a ground-breaking study described the gene expression-based subgroup classification of breast cancer [1]. Furthermore, a series of studies provided clarity that breast cancer can be categorized into at least five subtypes based on gene expression patterns. A tendency of individualized decisions for each group has been observed among clinicians for the treatment. Considering the changes in data presented in the last 40 years at St. Gallen Consensus Conferences, the main breast cancer treatment has transformed from surgical methods (based on anatomical information) to medical therapies (based on biological information) [2] This implied that the biological information of patients with breast cancer is now recognized as the primary concern.

In the 8^th^ edition of the American Joint Committee on Cancer (AJCC) in 2017, in addition to the anatomic stage, the importance of biology was included in the prognostic stage. Biological markers, such as histological grade, estrogen-receptor (ER), progesterone-receptor (PR), human epidermal growth factor receptor 2 (HER2), and nuclear protein Ki-67 status, have been used for tumor staging. Moreover, the expression levels of genes, such as hormone receptor (HR) -positive, are included [3]. According to the National Comprehensive Cancer Network (NCCN) guidelines version 3 in 2022, clinicians can decide to perform adjuvant chemotherapy or include ovarian function suppression (OFS) using the results of Oncotype Dx (ODX) assay for premenopausal patients with pathological node 0 (pN0) and tumor size >0.5 cm [4]. Clinicians can consider a similar approach even in pN positive (pN1) patients, while deciding the optimal treatment for the extension of the endocrine therapy for the following 5 years. Hence, each clinician needs to decide on treatment modalities, such as performing the multigene assay and adjuvant chemotherapy or adding OFS, considering adverse effects and risk of recurrence on switching or extending adjuvant endocrine therapy. While current guidelines offer predefined options, yet the inclusion of the term "consider" acknowledges the existence of certain conditions where making definitive decisions becomes challenging.

Due to the complexities associated with adjuvant endocrine therapy in recent times, clinicians can select various other available options by evaluating the clinical and genomic risks and potential benefits of each treatment. The Adjuvant! Online tool suggests the clinical risks using the information of histologic grade, the status of nodal metastases, and tumor size, and the genomic risk information can be obtained from prior studies, such as TAILORx, MINDACT, and RxPONDER trials [5–7]. Previous studies have demonstrated no benefit of additional adjuvant chemotherapy in postmenopausal women or patients over 50 years of age, whereas a 5–6% benefit was observed in premenopausal women or patients below 50 years of age. However, whether a combination of OFS with an aromatase inhibitor (AI) can be used in place of adjuvant chemotherapy (with ODX recurrence score [RS] of 16 or 21, to 25) in premenopausal women or patients with a low risk in the MammaPrint (MMP) test remains unclear.

Therefore, in this study, a survey was conducted to obtain the opinion of clinicians regarding the progress of decision-making on adjuvant therapy in premenopausal women. This study aimed to determine the tendency of adjuvant therapy in clinical practice.

## Materials and methods

We performed an online survey twice for clinicians who were members of the Korean Breast Cancer Society (KBCS). The questionnaire was delivered to each member via email from April to May 2022. In total, 92 clinicians replied with full answers. The survey aimed to collect information on the mean number of new patients that the clinicians had encountered per month. The survey included a questionnaire consisting of 62 items with multiple choices, and most of the questions were related to decision-making for different conditions and the adjuvant medical therapy selected in premenopausal patients. The questionnaire had details of patients with invasive ductal carcinoma with histologic, nuclear grade 2, and similar hormonal receptor conditions (ER/PR/HER2 as 8/8/1) but with different ages (35 years or 47 years), pathologic T and N stages, Ki-67 status, and/or the results of gene expression assays, such as 21-gene ODX or 70-gene MMP. The questions were designed to obtain information on the administration of adjuvant chemotherapy, clinicians’ choice of adjuvant endocrine therapy, and optimal duration for each endocrine therapy. Additional details are provided in the Supplementary Tables (available online). The statistical analysis employed the chi-square test to examine the statistical significance of response variations based on the career experience of specialists. For the purpose of comparing career experience, a distinction was made between specialists with below 16 years of experience and those with above 16 years, utilizing the mean value of 16 as the designated threshold. Statistical significance level was *p* < 0.05 bilateral. All statistical analyses were performed using the SPSS Software version 21.0 (IBM Corp., Armonk, USA).

Before starting the survey, we provided all clinicians who participated in the survey with the informed consent prior to completing the questionnaire, which contained summary of the purpose, the participants’ rights, and the confidentiality of their responses in online form. We proceeded beyond the consent form with all participants agreed with the information mentioned above. All procedures performed in studies involving human participants were in accordance with the ethical standards of the institutional and/or national research committee and with the 1964 Helsinki Declaration and its later amendments or comparable ethical standards.

## Results

**Fig 1:**
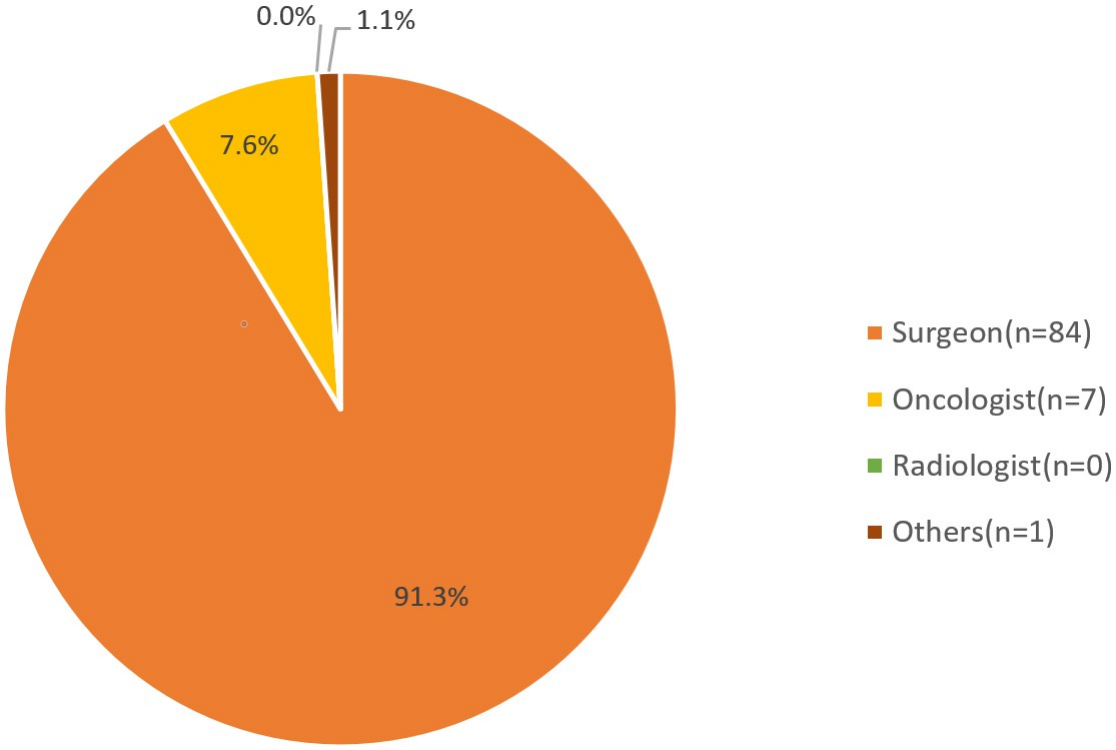
Specialty of clinicians who provided full responses. A total of 92 clinicians responded to the survey; among these, 91.3% (84/92) were breast surgeons, and 7.6% (7/92) were oncologists. We did not receive a reply from any radiologist.

A total of 92 clinicians responded to the survey; 91.3% were breast surgeons (84/92, Fig 1), and they received a specialist license (with their major) between 1978 and 2021 (S1 Fig in S1 File). Among clinicians included in the study, 64.1% (59/92) had an experience as a specialist of 16 years or below, while 35.9% (33/92) had a tenure exceeding 16 years. In response to the question on the number of new patients attended by clinicians per month, 82.6% of clinicians responded that they met less than 30 patients (with specific characteristics described in the Methods section) per month (S1 Table in S1 File).

In response to the first question on the frequency of adjuvant chemotherapy in patients with pT1N0, Ki-67 10%, and without an ODX result, 50.7% and 35.6% clinicians decided to perform adjuvant chemotherapy for 35 and 47 years old patients, respectively. For 35-year-old patients with pT2N0, Ki-67 50%, and without an ODX result, 96.8% of clinicians selected adjuvant chemotherapy. In addition, for the patient with ODX RS 16 and 21, with all other conditions remaining the same, 49.1% and 84.3% of clinicians preferred adjuvant chemotherapy, respectively. In 35-year-old patients with pT1N1, Ki-67 10%, and MMP low-risk status, only 41.7% of clinicians decided to perform adjuvant chemotherapy. In terms of age, when comparing individuals with similar conditions, younger individuals tended to opt for adjuvant chemotherapy more frequently. Furthermore, among individuals of the same age group, those with higher T-N staging or Ki-67 indices were more likely to have adjuvant chemotherapy (Table 1).

**Table 1:** Responses of clinicians regarding the decision on adjuvant chemotherapy for premenopausal women.

Question	Conditions	Options	Response (%)
Are you going to decide to do adjuvant chemotherapy for patients under these conditions?			
	1) 35-year-old, IDC, p**T1**N0, HG 2, NG 2, ER/PR/HER2 8/8/1, Ki-67 **10%,** ODX not performed	Yes	50.7
		No	49.3
	2) 35-year-old, IDC, p**T2**N0, HG 2, NG 2, ER/PR/HER2 8/8/1, Ki-67 **50%,** ODX not performed	Yes	96.8
		No	3.2
	3) 35-year-old, IDC, p**T2**N0, HG 2, NG 2, ER/PR/HER2 8/8/1, Ki-67 **50%,** ODX RS **16**	Yes	49.1
		No	50.9
	4) 35-year-old, IDC, p**T2**N0, HG 2, NG 2, ER/PR/HER2 8/8/1, Ki-67 **50%,** ODX RS **21**	Yes	84.3
		No	15.7
	5) 35-year-old, IDC, pT1**N1**, HG 2, NG 2, ER/PR/HER2 8/8/1, Ki-67 **10%**, **MMP low risk**	Yes	41.7
		No	58.3
	6) 47-year-old, IDC, pT1N0, HG 2, NG 2, ER/PR/HER2 8/8/1, Ki-67 10%, ODX not performed	Yes	35.6
		No	64.4

Abbreviations: Invasive ductal carcinoma (IDC), Oncotype Dx (ODX), recurrence score (RS), MammaPrint (MMP), estrogen-receptor (ER), progesterone-receptor (PR), human epidermal growth factor receptor 2 (HER2), nuclear grade (NG), histologic grade (HG)

In response to selecting adjuvant endocrine therapy after adjuvant chemotherapy in the first 5 years of treatment, 80.6% responded to implement tamoxifen (TMX) and/or OFS in 35-year-old patients with pT1N0, Ki-67 10%, and without ODX result. Further, 35.7% of responses were for a high likelihood of administering AI in combination with OFS for 5 years in 35-year-old patients with pT2N0, Ki-67 50%, and ODX RS 16. Notably, more clinicians (46.5%) decided to implement a combination of AI and OFS in patients with ODX RS 21, suggesting that fewer clinicians selected TMX with or without OFS. In 35-year-old patients with pT1N1, Ki-67 10%, and low risk in the MMP test, 55.0% of clinicians decided to administer AI in combination with OFS. Furthermore, 81.3% and 63.4% of clinicians used TMX with and without OFS in 47-year-old patients with pT1N0, Ki-67 10%, and without ODX and pT2N0, Ki-67 50%, and in those without ODX results, respectively. When it comes to determining adjuvant endocrine therapy following chemotherapy, among patients of the same age, a higher Ki-67 index and ODX RS were associated with a greater likelihood of selecting AI plus OFS. Moreover, among node-positive patients, there was an even higher proportion of clinicians opting for AI plus OFS (Table 2).

**Table 2:** Responses of clinicians regarding the decision on performing adjuvant endocrine therapy after adjuvant chemotherapy for premenopausal women.

Question	Conditions	Options	Response (%)
What is your choice of adjuvant endocrine therapy after chemotherapy for patients with each condition?			
	1) 35-year-old, IDC, pT1N0, HG 2, NG 2, ER/PR/HER2 8/8/1, Ki-67 10%, ODX not performed	TMX ± OFS, 5 years	80.6
		AI + OFS, 5 years	19.4
	2) 35-year-old, IDC, pT2N0, HG 2, NG 2, ER/PR/HER2 8/8/1, Ki-67 **50%,** ODX RS **16**	TMX ± OFS, 5 years	64.3
		AI + OFS, 5 years	35.7
	3) 35-year-old, IDC, pT2N0, HG 2, NG 2, ER/PR/HER2 8/8/1, Ki-67 **50%,** ODX RS **21**	TMX ± OFS, 5 years	53.5
		AI + OFS, 5 years	46.5
	4) 35-year-old, IDC, p**T1N1**, HG 2, NG 2, ER/PR/HER2 8/8/1, Ki-67 10%, **MMP low risk**	TMX ± OFS, 5 years	45.0
		AI + OFS, 5 years	55.0
	5) 47-year-old, IDC, pT1N0, HG 2, NG 2, ER/PR/HER2 8/8/1, Ki-67 10%, ODX not performed	TMX ± OFS, 5 years	81.3
		AI + OFS, 5 years	18.7
	6) 47-year-old, IDC, p**T2**N0, HG 2, NG 2, ER/PR/HER2 8/8/1, Ki-67 **50%,** ODX not performed	TMX ± OFS, 5 years	63.4
		AI + OFS, 5 years	36.6

Abbreviations: Invasive ductal carcinoma (IDC), Oncotype Dx (ODX), recurrence score (RS), MammaPrint (MMP), estrogen-receptor (ER), progesterone-receptor (PR), human epidermal growth factor receptor 2 (HER2), nuclear grade (NG), histologic grade (HG), tamoxifen (TMX), aromatase inhibitor (AI), ovarian function suppression (OFS)

Regarding clinicians’ choice of adjuvant endocrine therapy without prior chemotherapy, 62.9% of clinicians selected TMX with or without OFS for 35-year-old patients with pT1N0, Ki-67 10%, and without ODX result, whereas 53.6% selected this treatment for 35-year-old patients with pT2N0, Ki-67 50%, and ODX RS 16. Notably, for the same patient with ODX RS 21, more clinicians (85.7%) selected AI in combination with OFS. For 35-year-old patients with pT1N1 status and demonstrating a low-risk MMP profile, 57.1% of clinicians selected TMX with or without OFS. Furthermore, for 47-year-old patients with pT1N0, Ki-67 10%, and no ODX result, 75.9% responded with TMX with or without OFS. In cases which adjuvant chemotherapy was not administered, a similar trend was observed in determining adjuvant endocrine therapy, as shown in Table 2. However, it was noted that a higher proportion of clinicians opted for AI plus OFS for patients without prior chemotherapy, compared to those who received adjuvant chemotherapy under the same conditions (Table 3).

**Table 3:** Responses of clinicians regarding the decision on performing adjuvant endocrine therapy without prior adjuvant chemotherapy for premenopausal women.

Question	Conditions	Options	Response (%)
What is your choice of adjuvant endocrine therapy without prior chemotherapy for patients with each condition?			
	1) 35-year-old, IDC, pT1N0, HG 2, NG 2, ER/PR/HER2 8/8/1, Ki-67 10%, ODX not performed	TMX ± OFS, 5 years	62.9
		AI + OFS, 5 years	37.1
	2) 35-year-old, IDC, pT2N0, HG 2, NG 2, ER/PR/HER2 8/8/1, Ki-67 **50%,** ODX RS **16**	TMX ± OFS, 5 years	53.6
		AI + OFS, 5 years	46.4
	3) 35-year-old, IDC, p**T2**N0, HG 2, NG 2, ER/PR/HER2 8/8/1, Ki-67 50%, ODX RS **21**	TMX ± OFS, 5 years	14.3
		AI + OFS, 5 years	85.7
	4) 35-year-old, IDC, p**T1N1**, HG 2, NG 2, ER/PR/HER2 8/8/1, Ki-67 10%, **MMP low risk**	TMX ± OFS, 5 years	57.1
		AI + OFS, 5 years	42.9
	5) 47-year-old, IDC, pT1N0, HG 2, NG 2, ER/PR/HER2 8/8/1, Ki-67 10%, ODX not performed	TMX ± OFS, 5 years	75.9
		AI + OFS, 5 years	24.1

Abbreviations: Invasive ductal carcinoma (IDC), Oncotype Dx (ODX), recurrence score (RS), MammaPrint (MMP), estrogen-receptor (ER), progesterone-receptor (PR), human epidermal growth factor receptor 2 (HER2), nuclear grade (NG), histologic grade (HG), tamoxifen (TMX), aromatase inhibitor (AI), ovarian function suppression (OFS)

For questions related to the addition of OFS with TMX with and without prior adjuvant chemotherapy, 80.8% and 91.0% of clinicians responded to adding OFS with TMX after chemotherapy in 35-year-old patients with pT1N0, Ki-67 10%, and without ODX result and pT2N0 and Ki-67 50%, respectively. However, in 47-year-old patients with pT1N0, Ki-67 10%, and no ODX result, 69.2% of clinicians decided to not add OFS (Table 4A). In 35-year-old patients with pT1N0, Ki-67 10%, and without ODX result, 85.0% of respondents decided to add OFS in combination with TMX without prior chemotherapy, whereas only 54.6% replied to add OFS for 47-year-old patients with a similar profile (Table 4B). Among patients who received chemotherapy under identical conditions, a discernable inclination was observed towards less frequent addition of OFS. Moreover, even among patients who did not receive adjuvant chemotherapy, the addition rate of OFS did not exhibit a substantially higher tendency among comparatively older patients within the same conditions.

**Table 4:** Responses of clinicians regarding the decision on adding ovarian function suppression and tamoxifen with and without prior chemotherapy for premenopausal women.

Question	Conditions	Options	Response (%)
A. Are you going to decide to add OFS with Tamoxifen after chemotherapy?			
	1) 35-year-old, IDC, pT1N0, HG 2, NG 2, ER/PR/HER2 8/8/1, Ki-67 10%, ODX not performed	Add OFS	80.8
		No addition of OFS	19.2
	2) 35-year-old, IDC, p**T2**N0, HG 2, NG 2, ER/PR/HER2 8/8/1, Ki-67 **50%,** ODX not performed	Add OFS	91.0
		No addition of OFS	9.0
	3) 47-year-old, IDC, pT1N0, HG 2, NG 2, ER/PR/HER2 8/8/1, Ki-67 10%, ODX not performed	Add OFS	30.8
		No addition of OFS	69.2
B. Are you going to decide to add OFS with Tamoxifen without prior chemotherapy?			
	1) 35-year-old, IDC, pT1N0, HG 2, NG 2, ER/PR/HER2 8/8/1, Ki-67 10%, ODX not performed	Add OFS	85.0
		No addition of OFS	15.0
	2) 47-year-old, IDC, pT1N0, HG 2, NG 2, ER/PR/HER2 8/8/1, Ki-67 10%, ODX not performed	Add OFS	54.6
		No addition of OFS	45.4

Abbreviations: Invasive ductal carcinoma (IDC), Oncotype Dx (ODX), recurrence score (RS), MammaPrint (MMP), estrogen-receptor (ER), progesterone-receptor (PR), human epidermal growth factor receptor 2 (HER2), nuclear grade (NG), histologic grade (HG), tamoxifen (TMX), aromatase inhibitor (AI), ovarian function suppression (OFS)

Statistically significant difference was observed in the responses based on the career experience of specialists, in their opinions regarding the administration of adjuvant chemotherapy for a 35-year-old patient with pT1N0, ER/PR/HER2 8/8/1, Ki-67 10%, and without the use of ODX assay (*p*-value = 0.039). It was observed that clinicians with a tenure exceeding 16 years had a greater preference for choosing adjuvant chemotherapy for this patient group, compared to clinicians with less tenure. However, no meaningful differences were found for the remaining questions of from the whole survey (S2 Table in S1 File).

**Fig 2:**
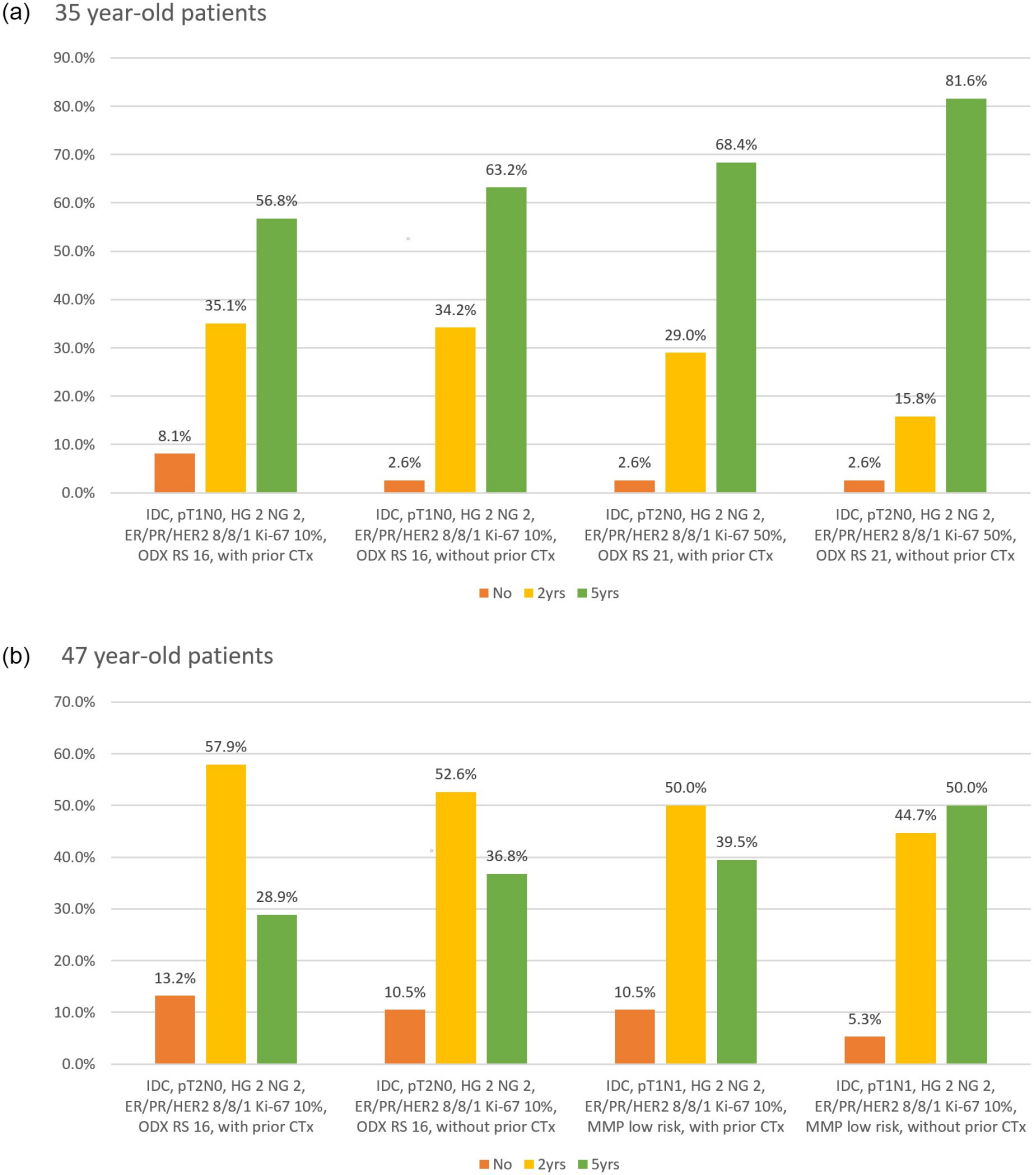
Duration of additional ovarian function suppression (OFS) in combination with tamoxifen for premenopausal women with or without adjuvant chemotherapy. The x-axis represents each condition of the virtual patient, whereas the y-axis represents the response percentages. (a) Duration of additional OFS in 35-year-old patients with or without adjuvant chemotherapy; 56.8% of clinicians selected to include OFS for 5 years for pT1N0, Ki-67 10%, Oncotype Dx recurrence score (ODX RS) 16, whereas 63.2% selected to include OFS for 5 years for patients without prior chemotherapy under equal pathological conditions. At a higher Ki-67 grade with ODX RS 21, 68.4% and 81.6% of clinicians selected to include OFS for 5 years with and without prior chemotherapy, respectively. (b) Duration of additional OFS in 47-year-old patients with or without adjuvant chemotherapy; 57.9% of clinicians selected to include OFS for only 2 years for pT2N0, Ki-67 10%, ODX 16, whereas 52.6% selected to include OFS for patients without prior chemotherapy under same pathological conditions. Further, 50.0% selected to include OFS for 2 years for pT1N1, Ki-67 10%, and low-risk profile from the MammaPrint assay and finished adjuvant chemotherapy in advance, whereas 50.0% answered to add OFS for 5 years without prior chemotherapy under same pathological condition.

The survey also consisted of questions on the duration of OFS in combination with TMX with and without prior adjuvant chemotherapy. For 35-years-old patients with pT1N0, Ki-67 10%, and ODX RS 16, more than half of clinicians answered to add OFS for 5 years, whereas more clinicians answered to add OFS for 5 years in patients of a similar pathologic profile without prior chemotherapy. Furthermore, in patients with a higher Ki-67 grade and ODX RS 21, 68.4% and 81.6% of clinicians replied to add OFS for 5 years with and without prior chemotherapy, respectively (Fig 2a). In 47-year-old patients with pT2N0, Ki-67 10%, and ODX 16 and who underwent prior chemotherapy, 57.9% of clinicians replied to add OFS for only 2 years. This percentage decreased in patients of similar pathologic profiles but without prior chemotherapy (52.6%). Half of the clinicians responded to add OFS for 2 years in patients with pT1N1, Ki-67 10%, and low risk in MMP assay and who finished adjuvant chemotherapy, whereas the other half answered to add OFS for 5 years in patients of similar pathologic profile but without prior chemotherapy (Fig 2b).

**Fig 3:**
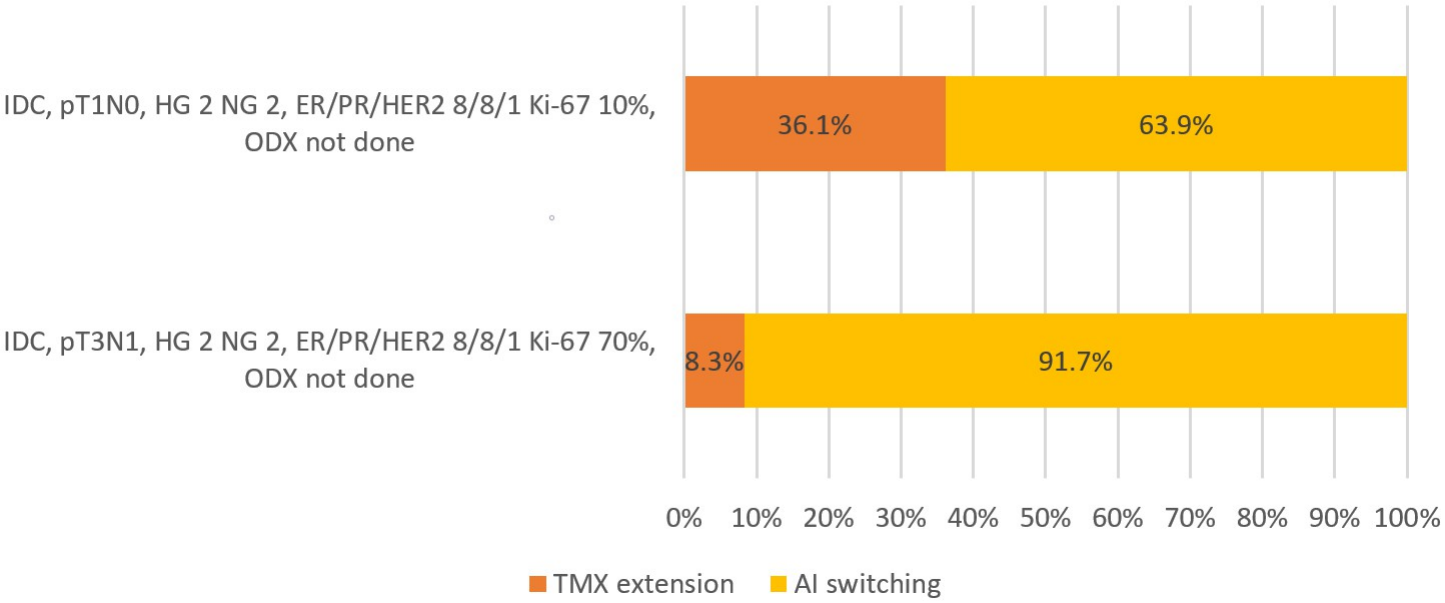
Decision on tamoxifen (TMX) extension versus switching to aromatase inhibitor (AI) for patients with a change of menopausal status after 5-year-use of TMX (47-years-old). For 47-year-old patients with a change of menopausal status after 5-year-use of TMX, 63.9% answered to switch from TMX to AI under the pathological condition of pT1N0 and Ki-67 10% with no ODX result, whereas 91.7% answered to switch from TMX to AI with the pathological condition of pT3N1 and Ki-67 70% without ODX result.

In response to the question of the decision of TMX extension versus switch to AI in 47-year-old patients with a change of menopausal status after 5-year-use of TMX, only 63.9% decided to switch from TMX to AI in the presence of pT1N0, Ki-67 10%, and with no ODX result profile; however, 91.7% preferred TMX to AI switch in patients with pT3N1, Ki-67 70%, and without an ODX result (Fig 3).

## Discussion

The transformation from anatomy to biology seems to be the central issue in contemporary breast cancer treatment. A typical case below demonstrates the importance of biological information in staging and making proper clinical decisions. A 56-year-old postmenopausal patient underwent a left breast cancer surgery, with pT2N0 as the final pathologic stage. The anatomic stage of the patient was stage IIA but the prognostic stage was IIIA, and the biological information was grade 3, ER positive, PR negative, and HER2 negative. After obtaining the data of ODX RS = 8, the prognostic stage was changed to IA, and the final adjuvant treatment for this patient was adjuvant endocrine therapy (letrozole alone). However, the implementation of individualized adjuvant therapy considering not only clinical risks but also biological risks for premenopausal, HR-positive, and HER2-negative breast cancer patients remains controversial. Hence, this study was performed to understand clinicians’ approaches in practice.

First of all, many clinicians tend to opt for diverse treatment choices with patients considering each age, and seem to select more proactive treatments as the patients are younger in age. From the case-control study of Hinton et al., age seems to be a key predictor of breast cancer biologic heterogeneity and may be a stronger determinant of heterogeneity than menopausal status [8]. A retrospective study with breast cancer patients of Johns Hopkins Hospital, focused especially with luminal A type, patients younger than 40 years old showed lower 5-year DFS (disease-free survival) and DMFS (distant metastasis-free survival) compared to the 41–60 year age group [9]. Therefore we designed our survey comparing between two distinct conditions with age, 35-year-old and 47-year old.

According to previous studies, genomic assays help make breast cancer treatment “PRECISE”. Regarding the use of biologic risk assessment using the multigene assay for HR-positive and HER2-negative in premenopausal patients with breast cancer, well-known trials have been performed, such as TAILORx, MINDACT, and RxPONDER. The TAILORx trial demonstrated a chemotherapy benefit of 1.6% (at 9 years) with RS 16–20 and 6.5% with RS 21–25 in patients > 50 years of age and had node-negative early-stage breast cancer [5]. The American Society of Clinical Oncology (ASCO) 2019 recommended adjuvant chemotherapy for premenopausal patients with RS of 21–25. Further, a consideration of clinical risk with RS of 16–20 was recommended based on the results of the TAILORx trial [10]. The MINDACT trial showed no survival difference regardless of adjuvant chemotherapy in patients with low clinical risk and high genomic risk (HR-positive, 79% of node-negative, and 21% of 1 to 3 node-positive patients). The updated MINDACT results announced in 2021, with an exploratory analysis by age, showed no chemotherapy benefit at the 8-year distant metastasis-free survival (DMFS); 90.2% with chemotherapy versus 90.0% without chemotherapy for older women (hazard ratio [HR] 0.82). Additionally, the 8-year DMFS was 93.6% with chemotherapy versus 88.6% without chemotherapy for younger women (HR 0.54) [6, 11]. The RxPONDER multicenter phase 3 trial randomized patients with HR-positive, HER2-negative, pN1mi, pN1, and RS of 25 or less to endocrine therapy alone versus chemotherapy followed by endocrine therapy. The analyses were performed by dividing subgroups into premenopausal and postmenopausal patients. There was no survival difference between chemotherapy and chemo-endocrine therapy groups for postmenopausal patients, whereas a statistically significant difference was observed for premenopausal patients, demonstrating an absolute difference of 5.2% on the 5-year IDFS (p = 0.0004) [7, 12]. Hence, in the summary of these three trials, additional chemotherapy demonstrated no benefit for postmenopausal patients with endocrine therapy; however, a chemotherapy benefit of 5–6% was observed for premenopausal patients with additional endocrine therapy, considering the results of multigene assays. The ASCO guidelines have been updated considering these conclusions on biomarkers for adjuvant endocrine and chemotherapy in early-stage breast cancer. Importantly the guidelines suggested not using the MMP assay for premenopausal patients, particularly those with a low clinical risk [13]. However, in these three trials, OFS was used limitedly only in 13–20% of premenopausal women, with a late effect starting after 4 years in the MINDACT trial. Further, there was little information on the occurrence of a chemotherapy-induced OFS.

Notably, the survey in this study did not ask clinicians whether to perform a genomic assay for premenopausal patients. In the survey, we provided clinicians with the conditions that contained the results of the genomic assays. We found that clinicians considered both clinical and genomic risks. Among responders, 96.8% replied that they performed chemotherapy for pT2N0 grade 2 HR-positive HER2-negative Ki-67 50% 35-year-old patients, compared to 50.7% for pT1N0 Ki-67 10% without ODX result, and only 35.6% answered for adjuvant chemotherapy for older, premenopausal patients when other conditions were the same. Moreover, more clinicians chose to perform chemotherapy according to the ODX or MMP results implying high genomic risk; 84.3% would perform chemotherapy with ODX RS 21 while 49.1% answered with ODX RS 16.

Clinicians are required to decide the type or duration of endocrine therapy, especially for premenopausal patients after deciding whether to perform adjuvant chemotherapy. Ines et al. suggested risk stratification among premenopausal patients and recommended endocrine therapy for 5–10 years. However, for high-risk premenopausal patients, it is also recommended to add OFS but no evidence has been obtained for continuing OFS for >5 years [14]. Previously, SOFT and TEXT trials provided optimal endocrine therapy options based on various experiences. In the SOFT trial, patients received TMX for 5 years, or TMX plus OFS, or exemestane plus OFS, whereas, in the TEXT trial, patients received TMX plus OPS or exemestane plus OFS. TMX plus OFS showed improvement in both DFS and overall survival (OS) compared to TMX alone. Moreover, exemestane plus OFS was related to an improvement of the 8-year DFS compared with TMX plus OFS (86.8 vs. 82.8%; HR 0.77; p<0.001) but not with OS (93.4 vs. 93.3%; HR 0.98; p = 0.84) [15–17]. More recent analyses showed that the overall rate of 8-year freedom from distant recurrence was 91.1%, and an online tool was suggested for risk-benefit calculations using SOFT/TEXT trial data [18, 19]. In contrast, in the similar patients group and follow-up duration, AI plus OFS showed no benefit compared with TMX plus OFS in the Austrian Breast and Colorectal Cancer Study Group Trial 12 (ABCSG-12) [20].

In terms of the optimal OFS duration, 1,293 patients with preserved ovarian function after chemotherapy who received TMX for 5 years with or without 2 years of OFS showed improved DFS and OS after the addition of 2-year OFS usage compared with TMX alone in the Hormone-Sensitive Breast Cancer Who remain Premenopausal or Regain Vaginal Bleeding After Chemotherapy (ASTRRA) of the Korean Breast Cancer Study Group (KBCSG). The key difference between the SOFT/TEXT trial and the ASTRRA trial was the duration of OFS (5 years versus 2 years) [21, 22]. In ASCO 2022, in the updated analyses of ASTRRA patients at the 8-year follow-up, a 5.2% DFS benefit of adding OFS (2 years) to TMX was observed with an HR of 0.67. These results provided reference data and showed that the duration of OFS could be individualized considering individual patient-related factors. [23]. Despite these previous studies, the optimal timing of initiation, duration, or interruption and type of adjuvant OFS remain unknown. It is also unclear whether adjuvant TMX monotherapy is sufficient for very young patients with favorable pathologic features, regarding balancing adherence and preserving the quality of life during endocrine therapy.

In our study, for premenopausal patients with adjuvant chemotherapy, clinicians tended to choose TMX plus OFS over AI plus OFS for 5 years, with lower clinical and genomic risks. Regarding a higher clinical risk of pN1 with a lower genomic risk, slightly more clinicians selected AI plus OFS for 5 years rather than TMX plus OFS. In premenopausal patients without adjuvant chemotherapy, the tendency was to select AI plus OFS rather than TMX plus OFS under both clinical and genomic risks. Furthermore, when the survey asked whether to add OFS with patients who had already finished adjuvant chemotherapy and were about to start TMX, more clinicians added OFS to the treatment for 35-year-old patients, whereas fewer clinicians selected OFS for treatment in 47-year-old patients. When the same question was adjusted to patients who were starting TMX without prior adjuvant chemotherapy, far more clinicians selected the “to add OFS for both age groups” response. Moreover, the study showed a trend of longer duration of additional OFS to TMX for patients with higher clinical and genomic risks and without adjuvant chemotherapy. In terms of the duration of OFS between different age groups, more responses were in favor of shorter OFS duration in older patients.

While specialists may consider the most optimal adjuvant therapy for their patients based on current guidelines, in actual clinical practice, it’s common to present all feasible treatment options to patients. Treatment decisions are made after thorough discussions, taking into account individual patient circumstances and preferences, including social and economic factors. There are current guidelines providing predefined options, it is important to note that these guidelines cannot encompass all possible decisions due to the diverse range of possibilities, leading to careful consideration of individualized treatment for each patient. Returning to the main focus, the survey fulfilled the initial purpose, which was to evaluate the current trend of each participating clinician on adjuvant therapy usage for premenopausal patients in Korea. In fact, we conducted an analysis to investigate potential statistical differences in responses based on the career experiences of each specialist. However, only minimal statistical differences were observed during the survey. The limitation of this study was the small number of respondents. In addition, the lack of quality control evaluation conducted on the questionnaire during the survey was considered a limitation of the study. In planning subsequent survey research, it seems crucial to incorporate a well-designed quality control program to ensure data integrity and reliability. However, this was the first study to survey clinicians’ choices of adjuvant therapy for premenopausal, HR-positive, early breast cancer patients in Korea. Besides, the survey could serve as the foundational research material for subsequent optimized studies. We aim to gather integrated, comprehensive responses from clinicians in the future to allow them to discuss and define the optimal adjuvant therapy for premenopausal, HR-positive, HER2-negative, and early-staged patients with breast cancer.

## Conclusions

The decision-making process regarding adjuvant endocrine therapy should take into account each patient’s clinical risk, genomic risk, and age of each patient, and clinicians should consult with patients in advance about potential adverse effects and compliance concerns. Furthermore, additional prospective randomized clinical trials about the clinical outcomes and optimal duration of OFS are required to evaluate ideal adjuvant therapy for each premenopausal patient with breast cancer.

## Supporting information

S1 File (DOCX)
