# Supplementary material for: Survey of clinicians on the use of adjuvant therapy for premenopausal women with breast cancer
Source: PLoS One. 2023 Aug 17;18(8):e0290174. doi: 10.1371/journal.pone.0290174 (PMC10434887; doi:10.1371/journal.pone.0290174)
Supplement: S1 File — (DOCX) [file pone.0290174.s001.docx]

**Survey of clinicians on the use of adjuvant therapy for premenopausal women with breast cancer**

Short title: Clinicians’ survey on adjuvant therapy for premenopausal breast cancer patients

Young-Won Lee^1¶^, Sei-Hyun Ahn^2&^, Young-jin Lee^1&^, Tae-Kyung Yoo^1&^, Jisun Kim^1&^, Il Yong Chung^1&^, Hee Jeong Kim^1&^, Beom Seok Ko^1&^, Jong Won Lee^1&^, Byung Ho Son^1&^, Sae Byul Lee^1*^

^1^Division of Breast Surgery, Department of Surgery, University of Ulsan College of Medicine, Asan Medical Center, Seoul, Republic of Korea

^2^Department of Surgery, Ewha Womans University College of Medicine, Ewha Womans University Mokdong Hospital, Seoul, Republic of Korea

**^*^** Corresponding author

E-mail address: [newstar153@hanmail.net](mailto:newstar153@hanmail.net) (SBL)

^¶^ These authors contributed equally to this work.

^&^ These authors also contributed equally to this work.

**Supporting information**

Supplementary Table 1. All questionnaire of the survey

Supplementary Table 2. All responses based on career experiences of specialists

Supplementary Fig 1. Years of the license of responders


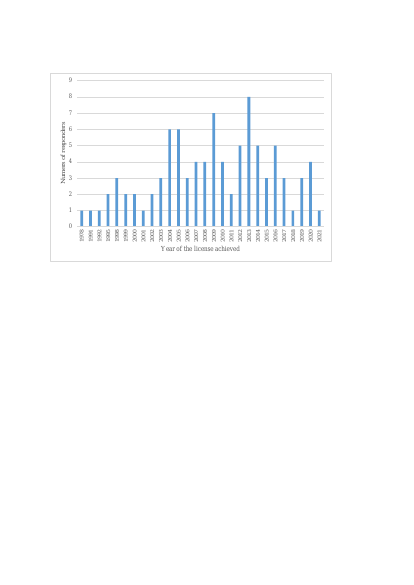


**Supplementary Table 1. All questionnaire of the survey**

| Questions | **Options** | **Response (%, n)** |
| --- | --- | --- |
| 1. What is your specialty? | - Surgeon - Oncologist - Radiologist - Others | - 91.3 (84) - 7.6 (7) - 0 (0) - 1.1 (1) |
| 1. How many new patients visit your office per month? | - 0−10 patients - 11−30 patients - 31−50 patients - more than 51 patients | - 42.4 (39) - 40.2 (37) - 7.6 (7) - 9.8 (9) |
| Condition 1: 35 years of age , IDC, pT1N0, HG 2, NG 2, ER/PR/HER2 8/8/1, Ki-67 10%, ODX not performed | | |
| 1. Do you intend to administer adjuvant chemotherapy to the patient with the condition described above? | - Yes - No | - 50.7 (39) - 49.3 (38) |
| 1. What is your choice of adjuvant endocrine therapy after chemotherapy for patient with the condition described above? | - TMX ± OFS, 5 years - AI + OFS, 5 years | - 80.6 (29) - 19.4 (7) |
| 1. What is your choice of adjuvant endocrine therapy without prior chemotherapy for patients with the condition described above? | - TMX ± OFS, 5 years - AI + OFS, 5 years | - 62.9 (22) - 37.1 (13) |
| 1. Are you going to decide to add OFS with TMX after chemotherapy? | - Yes - No | - 80.8 (21) - 19.2 (5) |
| 1. Are you going to decide to add OFS with TMX without prior chemotherapy? | - Yes - No | - 85.0 (17) - 15.0 (3) |
| Condition 2: 35 years of age, IDC, pT2N0, HG 2, NG 2, ER/PR/HER2 8/8/1, Ki-67 50%, ODX not performed | | |
| 1. Do you intend to administer adjuvant chemotherapy to the patient with the condition described above? | - Yes - No | - 96.7 (59) - 3.3 (2) |
| 1. What is your choice of adjuvant endocrine therapy after chemotherapy for patient with the condition described above? | - TMX ± OFS, 5 years - AI + OFS, 5 years | - 56.0 (33) - 44.0 (26) |
| 1. What is your choice of adjuvant endocrine therapy without prior chemotherapy for patients with the condition described above? | - TMX ± OFS, 5 years - AI + OFS, 5 years | - 100 (2) - 0 (0) |
| 1. Are you going to decide to add OFS with TMX after chemotherapy? | - Yes - No | - 91.0 (30) - 9.1 (3) |
| 1. Are you going to decide to add OFS with TMX without prior chemotherapy? | - Yes - No | - 100 (2) - 0 (0) |
| Condition 3: 35 years of age, IDC, pT2N0, HG 2, NG 2, ER/PR/HER2 8/8/1, Ki-67 50%, ODX RS 16 | | |
| 1. Do you intend to administer adjuvant chemotherapy to the patient with the condition described above? | - Yes - No | - 49.1 (27) - 50.9 (28) |
| 1. What is your choice of adjuvant endocrine therapy after chemotherapy for patient with the condition described above? | - TMX ± OFS, 5 years - AI + OFS, 5 years | - 64.3 (18) - 35.7 (10) |
| 1. What is your choice of adjuvant endocrine therapy without prior chemotherapy for patients with the condition described above? | - TMX ± OFS, 5 years - AI + OFS, 5 years | - 53.6 (15) - 46.4 (13) |
| 1. Are you going to decide to add OFS with TMX after chemotherapy? | - Yes - No | - 100 (14) - 0 (0) |
| 1. Are you going to decide to add OFS with TMX without prior chemotherapy? | - Yes - No | - 86.7 (13) - 13.3 (2) |
| Condition 4: 35 years of age, IDC, pT2N0, HG 2, NG 2, ER/PR/HER2 8/8/1, Ki-67 50%, ODX RS 21 | | |
| 1. Do you intend to administer adjuvant chemotherapy to the patient with the condition described above? | - Yes - No | - 84.3 (43) - 15.7 (8) |
| 1. What is your choice of adjuvant endocrine therapy after chemotherapy for patient with the condition described above? | - TMX ± OFS, 5 years - AI + OFS, 5 years | - 53.5 (23) - 46.5 (20) |
| 1. What is your choice of adjuvant endocrine therapy without prior chemotherapy for patients with the condition described above? | - TMX ± OFS, 5 years - AI + OFS, 5 years | - 14.3 (1) - 85.7 (6) |
| 1. Are you going to decide to add OFS with TMX after chemotherapy? | - Yes - No | - 95.7 (22) - 4.3 (1) |
| 1. Are you going to decide to add OFS with TMX without prior chemotherapy? | - Yes - No | - 100 (1) - 0 (0) |
| 1. How long will you administer OFS with TMX after chemotherapy? | - No addition of OFS - 2 years - 5 years | - 2.6 (1) - 29.0 (11) - 68.4 (26) |
| 1. How long will you administer OFS with TMX, without prior chemotherapy? | - No addition of OFS - 2 years - 5 years | - 2.6 (1) - 15.8 (6) - 81.6 (31) |
| Condition 5: 35 years of age, IDC, pT1N1, HG 2, NG 2, ER/PR/HER2 8/8/1, Ki-67 10%, MMP low risk | | |
| 1. Do you intend to administer adjuvant chemotherapy to the patient with the condition described above? | - Yes - No | - 41.7 (20) - 58.3 (28) |
| 1. What is your choice of adjuvant endocrine therapy after chemotherapy for patient with the condition described above? | - TMX ± OFS, 5 years - AI + OFS, 5 years | - 45.0 (9) - 55.0 (11) |
| 1. What is your choice of adjuvant endocrine therapy without prior chemotherapy for patients with the condition described above? | - TMX ± OFS, 5 years - AI + OFS, 5 years | - 57.1 (16) - 42.9 (12) |
| 1. Are you going to decide to add OFS with TMX after chemotherapy? | - Yes - No | - 100 (8) - 0 (0) |
| 1. Are you going to decide to add OFS with TMX without prior chemotherapy? | - Yes - No | - 93.3 (14) - 6.7 (1) |
| Condition 6: 47 years of age, IDC, pT1N0, HG 2, NG 2, ER/PR/HER2 8/8/1, Ki-67 10%, ODX not performed | | |
| 1. Do you intend to administer adjuvant chemotherapy to the patient with the condition described above? | - Yes - No | - 35.6 (16) - 64.4 (29) |
| 1. What is your choice of adjuvant endocrine therapy after chemotherapy for patient with the condition described above? | - TMX ± OFS, 5 years - AI + OFS, 5 years | - 81.3(13) - 18.7 (3) |
| 1. What is your choice of adjuvant endocrine therapy without prior chemotherapy for patients with the condition described above? | - TMX ± OFS, 5 years - AI + OFS, 5 years | - 75.9 (22) - 24.1 (7) |
| 1. Are you going to decide to add OFS with TMX after chemotherapy? | - Yes - No | - 30.8 (4) - 69.2 (9) |
| 1. Are you going to decide to add OFS with TMX without prior chemotherapy? | - Yes - No | - 54.6 (12) - 45.4 (10) |
| 1. Which do you prefer, TMX extension versus switching to AI, for the patient with condition described above, who has change of menopausal status after 5-year-use of TMX? | - TMX extension - AI switching | - 36.1 (13) - 63.9 (23) |
| Condition 7: 47 years of age, IDC, pT2N0, HG 2, NG 2, ER/PR/HER2 8/8/1, Ki-67 70%, ODX not performed | | |
| 1. Do you intend to administer adjuvant chemotherapy to the patient with the condition described above? | - Yes - No | - 95.4 (41) - 4.6 (2) |
| 1. What is your choice of adjuvant endocrine therapy after chemotherapy for patient with the condition described above? | - TMX ± OFS, 5 years - AI + OFS, 5 years | - 63.4 (26) - 36.6 (15) |
| 1. What is your choice of adjuvant endocrine therapy without prior chemotherapy for patients with the condition described above? | - TMX ± OFS, 5 years - AI + OFS, 5 years | - 100 (2) - 0 (0) |
| 1. Are you going to decide to add OFS with TMX after chemotherapy? | - Yes - No | - 57.7 (15) - 42.3 (11) |
| 1. Are you going to decide to add OFS with TMX without prior chemotherapy? | - Yes - No | - 50.0 (1) - 50.0 (1) |
| Condition 8: 47 years of age, IDC, pT2N0, HG 2, NG 2, ER/PR/HER2 8/8/1, Ki-67 50%, ODX RS 16 | | |
| 1. Are you going to decide to do adjuvant chemotherapy to patient with the condition described above? | - Yes - No | - 41.5 (17) - 58.5 (24) |
| 1. What is your choice of adjuvant endocrine therapy after chemotherapy for patient with the condition described above? | - TMX ± OFS, 5 years - AI + OFS, 5 years | - 70.6 (12) - 29.4 (5) |
| 1. What is your choice of adjuvant endocrine therapy without prior chemotherapy for patients with the condition described above? | - TMX ± OFS, 5 years - AI + OFS, 5 years | - 58.3 (14) - 41.7 (10) |
| 1. Are you going to decide to add OFS with TMX after chemotherapy? | - Yes - No | - 66.7 (8) - 33.3 (4) |
| 1. Are you going to decide to add OFS with TMX without prior chemotherapy? | - Yes - No | - 73.3 (11) - 26.7 (4) |
| Condition 9: 47 years of age, IDC, pT2N0, HG 2, NG 2, ER/PR/HER2 8/8/1, Ki-67 50%, ODX RS 21 | | |
| 1. Do you intend to administer adjuvant chemotherapy to the patient with the condition described above? | - Yes - No | - 73.2 (30) - 26.8 (11) |
| 1. What is your choice of adjuvant endocrine therapy after chemotherapy for patient with the condition described above? | - TMX ± OFS, 5 years - AI + OFS, 5 years | - 60.0 (18) - 40.0 (12) |
| 1. What is your choice of adjuvant endocrine therapy without prior chemotherapy for patients with the condition described above? | - TMX ± OFS, 5 years - AI + OFS, 5 years | - 54.6 (6) - 45.4 (5) |
| 1. Are you going to decide to add OFS with TMX after chemotherapy? | - Yes - No | - 66.7 (12) - 33.3 (6) |
| 1. Are you going to decide to add OFS with TMX without prior chemotherapy? | - Yes - No | - 50.0 (3) - 50.0 (3) |
| Condition 10: 47 years of age, IDC, pT1N1, HG 2, NG 2, ER/PR/HER2 8/8/1, Ki-67 10%, MMP low risk | | |
| 1. Do you intend to administer adjuvant chemotherapy to the patient with the condition described above? | - Yes - No | - 29.3 (12) - 70.7 (29) |
| 1. What is your choice of adjuvant endocrine therapy after chemotherapy for patient with the condition described above? | - TMX ± OFS, 5 years - AI + OFS, 5 years | - 84.6 (11) - 15.4 (2) |
| 1. What is your choice of adjuvant endocrine therapy without prior chemotherapy for patients with the condition described above? | - TMX ± OFS, 5 years - AI + OFS, 5 years | - 42.9 (12) - 57.1 (16) |
| 1. Are you going to decide to add OFS with TMX after chemotherapy? | - Yes - No | - 50.0 (5) - 50.0 (5) |
| 1. Are you going to decide to add OFS with TMX without prior chemotherapy? | - Yes - No | - 75.0 (9) - 25.0 (3) |
| 1. How long will you administer OFS with TMX after chemotherapy? | - No addition of OFS - 2 years - 5 years | - 10.5 (4) - 50.0 (19) - 39.5 (15) |
| 1. How long will you administer OFS with TMX, without prior chemotherapy? | - No addition of OFS - 2 years - 5 years | - 5.3 (2) - 44.8 (17) - 50.0 (19) |
| Condition 11: 35 years of age, IDC, pT1N0, HG 2, NG 2, ER/PR/HER2 8/8/1 Ki-67 10%, ODX RS 16 | | |
| 1. How long will you administer OFS with TMX after chemotherapy? | - No addition of OFS - 2 years - 5 years | - 8.1 (3) - 35.1(13) - 56.8 (21) |
| 1. How long will you administer OFS with TMX, without prior chemotherapy? | - No addition of OFS - 2 years - 5 years | - 2.6 (1) - 34.2 (13) - 63.2 (24) |
| Condition 12: 47 years of age, IDC, pT2N0, HG 2, NG 2, ER/PR/HER2 8/8/1 Ki-67 10%, ODX RS 16 | | |
| 1. How long will you administer OFS with TMX after chemotherapy? | - No addition of OFS - 2 years - 5 years | - 13.1(5) - 57.9 (22) - 29.0 (11) |
| 1. How long will you administer OFS with TMX, without prior chemotherapy? | - No addition of OFS - 2 years - 5 years | - 10.5 (4) - 52.6 (20) - 36.8 (14) |
| Condition 13: 47 years of age, IDC, pT3N1, HG 2, NG 2, ER/PR/HER2 8/8/1 Ki-67 70%, ODX not performed | | |
| 1. Which do you prefer, TMX extension or switching to AI, for the patient with the above-mentioned condition who has a change in menopausal status after a 5-year TMX treatment with the condition described above? | - TMX extension - AI switching | - 8.3 (3) - 91.7 (33) |

**Abbreviations:** Invasive ductal carcinoma (IDC), Oncotype Dx (ODX), recurrence score (RS), MammaPrint (MMP), estrogen-receptor (ER), progesterone-receptor (PR), human epidermal growth factor receptor 2 (HER2), nuclear grade (NG), histologic grade (HG), tamoxifen (TMX), aromatase inhibitor (AI), ovarian function suppression (OFS)

**Supplementary Table 2. All responses based on career experiences of specialists;
1) Responses regarding the decision on adjuvant chemotherapy for premenopausal women, 2) Responses regarding the decision on performing adjuvant endocrine therapy after adjuvant chemotherapy for premenopausal women, 3) Response of clinicians regarding the decision on performing adjuvant endocrine therapy without prior adjuvant chemotherapy for premenopausal women, 4) Response of clinicians regarding the decision on adding ovarian function suppression and tamoxifen with and without prior chemotherapy for premenopausal women**

**2-1) Responses based on career experiences of specialists, regarding the decision on adjuvant chemotherapy for premenopausal women**

| Question | Conditions | Options | Response (%),  based on career experiences | | *p-value* |
| --- | --- | --- | --- | --- | --- |
|  |  |  | ≤ 16 years | ＞ 16 years |  |
| Are you going to decide to do adjuvant chemotherapy for patients under these conditions? | | | | | |
|  | 1) 35-year-old, IDC, p**T1**N0, HG 2, NG 2, ER/PR/HER2 8/8/1, Ki-67 **10%,** ODX not performed | Yes | 42.0 | 66.7 | 0.039 |
|  |  | No | 58.0 | 33.3 |  |
|  | 2) 35-year-old, IDC, p**T2**N0,  HG 2, NG 2, ER/PR/HER2  8/8/1, Ki-67 **50%,** ODX not  performed | Yes | 97.6 | 95.0 | 0.598 |
|  |  | No | 2.4 | 5.0 |  |
|  | 3) 35-year-old, IDC, p**T2**N0, HG 2, NG 2, ER/PR/HER2 8/8/1, Ki-67 **50%,** ODX RS **16** | Yes | 52.6 | 41.2 | 0.684 |
|  |  | No | 47.4 | 58.8 |  |
|  | 4) 35-year-old, IDC, p**T2**N0, HG 2, NG 2, ER/PR/HER2 8/8/1, Ki-67 **50%,** ODX RS **21** | Yes | 85.7 | 81.3 | 0.684 |
|  |  | No | 14.3 | 18.7 |  |
|  | 5) 35-year-old, IDC, pT1**N1**, HG 2, NG 2, ER/PR/HER2 8/8/1, Ki-67 **10%**, **MMP low risk** | Yes | 39.4 | 46.7 | 0.826 |
|  |  | No | 60.6 | 53.3 |  |
|  | 6) 47-year-old, IDC, pT1N0, HG 2, NG 2, ER/PR/HER2 8/8/1, Ki-67 10%, ODX not performed | Yes | 36.7 | 33.3 | 0.826 |
|  |  | No | 63.3 | 66.7 |  |

**2-2) Responses based on career experiences of specialists, regarding the decision on performing adjuvant endocrine therapy after adjuvant chemotherapy for premenopausal women**

| Question | Conditions | Options | Response (%), based on career experiences | | *p-value* |  |
| --- | --- | --- | --- | --- | --- | --- |
|  |  |  | ≤ 16 years | ＞ 16 years |  |  |
| What is your choice of adjuvant endocrine therapy after chemotherapy for patients with each condition? | | | | | | |
|  | 1) 35-year-old, IDC, pT1N0, HG 2, NG 2, ER/PR/HER2 8/8/1, Ki-67 10%, ODX not performed | TMX ± OFS, 5 years | 75.0 | 87.5 | 0.346 |  |
|  |  | AI ± OFS, 5 years | 25.0 | 12.5 |  |  |
|  | 2) 35-year-old, IDC, pT2N0, HG 2, NG 2, ER/PR/HER2 8/8/1, Ki-67 **50%,** ODX RS **16** | TMX ± OFS, 5 years | 61.9 | 71.4 | 0.649 |  |
|  |  | AI + OFS, 5 years | 38.1 | 28.6 |  |  |
|  | 3) 35-year-old, IDC, pT2N0, HG 2, NG 2, ER/PR/HER2 8/8/1, Ki-67 **50%,** ODX RS **21** | TMX ± OFS, 5 years | 50.0 | 61.5 | 0.486 |  |
|  |  | AI + OFS, 5 years | 50.0 | 38.5 |  |  |
|  | 4) 35-year-old, IDC, p**T1N1**, HG 2, NG 2, ER/PR/HER2 8/8/1, Ki-67 10%, **MMP low risk** | TMX ± OFS, 5 years | 46.2 | 42.9 | 0.888 |  |
|  |  | AI + OFS, 5 years | 53.8 | 57.1 |  |  |
|  | 5) 47-year-old, IDC, pT1N0, HG 2, NG 2, ER/PR/HER2 8/8/1, Ki-67 10%, ODX not performed | TMX ± OFS, 5 years | 81.8 | 80.0 | 0.931 |  |
|  |  | AI + OFS, 5 years | 18.2 | 20.0 |  |  |
|  | 6) 47-year-old, IDC, p**T2**N0, HG 2, NG 2, ER/PR/HER2 8/8/1, Ki-67 **50%,** ODX not performed | TMX ± OFS, 5 years | 66.7 | 57.1 | 0.548 |  |
|  |  | AI + OFS, 5 years | 33.3 | 42.9 |  |  |

**2-3) Responses based on career experiences of specialists, regarding the decision on performing adjuvant endocrine therapy without prior adjuvant chemotherapy for premenopausal women**

| Question | Conditions | Options | Response (%), based on career experiences | | *p-value* | |
| --- | --- | --- | --- | --- | --- | --- |
|  |  |  | ≤ 16 years | ＞ 16 years |  |  |
| What is your choice of adjuvant endocrine therapy without prior chemotherapy for patients with each condition? | | | | | |  |
|  | 1) 35-year-old, IDC, pT1N0, HG 2, NG 2, ER/PR/HER2 8/8/1, Ki-67 10%, ODX not performed | TMX ± OFS, 5 years | 59.3 | 75.0 | 0.418 | |
|  |  | AI + OFS, 5 years | 40.7 | 25.0 |  |  |
|  | 2) 35-year-old, IDC, pT2N0, HG 2, NG 2, ER/PR/HER2 8/8/1, Ki-67 **50%,** ODX RS **16** | TMX ± OFS, 5 years | 55.6 | 50.0 | 0.778 | |
|  |  | AI + OFS, 5 years | 44.4 | 50.0 |  |  |
|  | 3) 35-year-old, IDC, p**T2**N0, HG 2, NG 2, ER/PR/HER2 8/8/1, Ki-67 50%, ODX RS **21** | TMX ± OFS, 5 years | 20.0 | 0.0 | 0.495 | |
|  |  | AI + OFS, 5 years | 80.0 | 100.0 |  |  |
|  | 4) 35-year-old, IDC, p**T1N1**, HG 2, NG 2, ER/PR/HER2 8/8/1, Ki-67 10%, **MMP low risk** | TMX ± OFS, 5 years | 60.0 | 50.0 | 0.629 | |
|  |  | AI + OFS, 5 years | 40.0 | 50.0 |  |  |
|  | 5) 47-year-old, IDC, pT1N0, HG 2, NG 2, ER/PR/HER2 8/8/1, Ki-67 10%, ODX not performed | TMX ± OFS, 5 years | 73.7 | 80.0 | 0.706 | |
|  |  | AI + OFS, 5 years | 26.3 | 20.0 |  |  |

**2-4) Responses based on career experiences of specialists, regarding the decision on adding ovarian function suppression and tamoxifen with and without prior chemotherapy for premenopausal women**

| Question | Conditions | Options | Response (%), based on career experiences | | | *p-value* | |  |
| --- | --- | --- | --- | --- | --- | --- | --- | --- |
|  |  |  | ≤ 16 years | ＞ 16 years | |  |  |  |
| 1. Are you going to decide to add OFS with Tamoxifen after chemotherapy? | | | | | | | | |
|  | 1) 35-year-old, IDC, pT1N0, HG 2, NG 2, ER/PR/HER2 8/8/1, Ki-67 10%, ODX not performed | Add OFS | 85.7 | | 75.0 | | 0.490 | |
|  |  | No addition of OFS | 14.3 | | 25.0 | |  |  |
|  | 2) 35-year-old, IDC, p**T2**N0, HG 2, NG 2, ER/PR/HER2 8/8/1, Ki-67 **50%,** ODX not performed | Add OFS | 91.3 | | 90.0 | | 0.905 | |
|  |  | No addition of OFS | 8.7 | | 10.0 | |  |  |
|  | 3) 47-year-old, IDC, pT1N0, HG 2, NG 2, ER/PR/HER2 8/8/1, Ki-67 10%, ODX not performed | Add OFS | 44.4 | | 0.0 | | 0.109 | |
|  |  | No addition of OFS | 55.6 | | 100.0 | |  |  |
| 1. Are you going to decide to add OFS with Tamoxifen without prior chemotherapy? | | | | | | | | |
|  | 1) 35-year-old, IDC, pT1N0, HG 2, NG 2, ER/PR/HER2 8/8/1, Ki-67 10%, ODX not performed | Add OFS | 87.5 | | 75.0 | | 0.531 | |
|  |  | No addition of OFS | 12.5 | | 25.0 | |  |  |
|  | 2) 47-year-old, IDC, pT1N0, HG 2, NG 2, ER/PR/HER2 8/8/1, Ki-67 10%, ODX not performed | Add OFS | 57.1 | | 50.0 | | 0.746 | |
|  |  | No addition of OFS | 42.9 | | 50.0 | |  |  |
